# Hemiopia

**Published:** 1877-10

**Authors:** Wm. Dickinson

**Affiliations:** No. 620 Locust St.; St. Louis, Mo.


### HEMIOPIA.

By Wm. Dickinson, M.D., St. Louis, Mo.

Hemiopia is comparatively a rare affection: it is not a disease; it is but a symptom of disease. This term signifies half-vision; that is, vision with only one-half of the eye, the other half being insensible to visual impressions; implying that condition of the rods and cones, or of the optic nerve-fibres of the retina, or of the optic-nerve itself, or of the optic tract, in which only one-half of these several portions is capable of receiving or transmitting visual impressions to their destination where they are converted into perceptions. The lesion determining this condition may exist in one eye only, or in both; generally, however, the *right* or the *left half* of all objects is the portion unseen; the upper or the lower halves may be thus affected, though rarely. Again, the temporal half of each retina may be the portion insensible. According to the generally received doctrine of the semi-decussation (of which there are grave doubts) of the optic nerves, this phenomenon is to be explained by lesion of those fibres which do not cross at the chiasma. In such cases the impairment of vision is often very rapid, the sight being utterly destroyed within a few days. Usually, however, both eyes are symmetrically affected, the right-half of each retina, or the left-half of each retina, being involved. In such cases the hemiopia is termed equilateral or homonymous. This condition may exist alone, uncomplicated with any other affection; it is then transient and induced by reflex action, the result of the perverted function of some remote organ. It is often found associated with hemiplegia; then it becomes an infallible index of a disease, which even now sapping, will, unless arrested, ultimately destroy the citadel of life.

Hemiopia sometimes supervenes slowly, and is then due to softening of portions of the brain; but more frequently it is sudden in its invasion and is dependent upon hemorrhagic or serous effusion. If this is in small quantity and speedily absorbed, the affection will soon disappear; if it be of functional or reflex origin, it will subside with the disappearance of the cause. These recurrences and intermissions, in long series, may characterize its visitations.

Dr. Wollaston, who, in the year 1824, directed attention to this affection by his paper on the semi-decussation of the optic nerves, himself suffered two attacks of this affection, and has left on record his experiences. The second attack recurred after an interval of twenty years; but the effects, *apparently* to him, entirely disappeared after fifteen or twenty minutes. In the first attack the left half of all objects appeared dark; and in the second attack, the right half. The first attack he attributed to violent exercise which he had taken two hours before. He states, he suddenly found he could see but one-half the face of a man whom he met; and it was the same with respect to every object looked at. For instance, in attempting to read the name, Johnson, over a door he saw only *son*, the commencement of the name being wholly obliterated from his view. The second attack was without any assignable cause. Judging from the narrative whence this extract is taken, it is evident the Doctor had but an imperfect appreciation of the real cause of his hemiopia. For having died four years later, an examination of his brain revealed a condition of which probably he had no suspicion. The right thalamus was of unusually large size, and little or no vestige of its natural substance was perceptible. It had been converted into a tumor, harder than the brain itself, somewhat of a caseous substance towards the circumference, and in the centre of a brown color, soft and in a half-dissolved state. The right optic-nerve, where it passes on the outside of the optic thalamus, was also of a brown color, more expanded and softer than natural.

In harmony with the theory of the semi-decussation of the optic nerves, is the description of these nerves given by "Wilson" in his Anatomy, to which reference may be made.

With this introduction we pass to the narrative of the case by which it was suggested.

S. R. is a man of about forty years of age, of temperate habits, light complexion, hair and eyes; of medium height, and weight about 160 pounds; his vocation that of a merchant, and for several years has closely devoted himself to business often to a late hour of the night. He states that scrofula is a hereditary peculiarity of his family, and that his father died suddenly while at church, at the age of sixty-two years. He confesses himself to have been the subject of syphilis twenty years since, but was then cured and no evidence of the disease has since recurred. In July, of '73, during a period of good health, though the subject of frequent headaches, while engaged in the ordinary routine of business at the store, he suddenly experienced a partial loss of motion and sensation of his left side and extremities (partial .hemiplegia) without apparent cause of any kind. From this attack he recovered in the course of a week or ten days, and resumed his business. During the succeeding three years he was exempt from any recurrence of a similar character. During July, of '76, though consciously ill, he yet attended to business as usual, which was not necessarily confining or requiring much mental effort. One morning, while walking to his store, less than one-half mile distant, he experienced a sensation of numbness of the left side, involving face, arm, body and leg—losing the use of the latter to such a degree as to induce him to sit down. After a little time he so far recovered as to be able to reach the store; but after two or three hours he went home in a carriage. During this attack he observed some formication in the extremities, but no pricking sensations and no distortion of the face or mouth. He has never noticed any differences in temperature of his two sides; never lost entire control of use of left arm, hand or leg, though they were perceptibly weaker than those of the other side. His gait, at times, was a little unsteady and often to such degree as to require him to sit. He has never suffered from constipation, never had a fall or received a blow upon his head. His hearing during the past year has diminished in a slight degree, though yet good.

His medical adviser prescribed appropriate remedies, and recommended a period of travel for respite from business and for recreation. This advice was adopted and much benefit received. About Sept. 20th, he first observed he was unable to see distinctly objects on the left side of the median line. This inability has since much increased. He has also observed some confusion of ideas and some difficulty in concentrating his mind. He has also perceived some difficulty in articulating distinctly. His wife has noticed occasional twitching of of the eye and an obvious change in his manner and general deportment—a peculiarity also observed by his friends,—and in his disposition a degree of restlessness and irritability; his mental equilibrium being disturbed by circumstances which formerly produced no effect, often quite excited by those of a trivial nature; but of all these latter mental deviations he himself is totally unconscious. He has also experienced a sensation of fullness in his throat, as if it was swollen, but differing from that condition consequent upon an attack of bronchitis. Respiration is somewhat embarrassed and deglutition also, but the sense of taste is not perceptibly affected. For about a year he has observed that his vision was not quite as acute as formerly. He has, to some extent, used tobacco by smoking, but not intoxicating liquors. Recently his feet have been almost habitually cold, while formerly they were disagreeably warm, amounting to a hot sensation when touched.

Such was the general condition of our patient when he first consulted me. In his manner there was nothing specially observable, save perhaps a semblance of listlessness or of general lassitude; in all other respects he bore the appearance of a man in perfect health. The action and sounds of the heart were normal ; but during the past year he has observed that active exertion, or to an unwonted degree has produced a hurried respiration from which he was formerly free. The functions of all the other organs, thoracic and abdominal, are normally performed, and has now perfectly recovered motion and sensation of the left side.

In respect to the condition of his eyes, there was nothing worthy of note excepting the slight amblyopia and the hemiopia. Two objects held before his eyes at fifteen inches distance, are distinctly seen by both eyes; upon fixing vision upon the left (to him) object, and moving the right object to his right, it is seen throughout the normal range; but fixing vision as before, upon the right object, (as presented to him) and removing the left object to his left, for the first three and a half inches it is seen, it then seems to enter a shadow, a penumbra, which appears gradually to increase in density as the object moves, till it entirely disappears. With the ophthalmoscope, all the dioptric media were found transparent, the details of the fundus distinctly seen and clearly defined, and the vessels of normal size and appearance. No glasses seemed to ameliorate the amblyopia.

The ophthalmascope demonstrates that the retina is not at fault. The patient states that only *one half* of objects are unseen, and that this blindness exists on the same side in both eyes. Anatomy, according to the generally accepted (not indubitably proved) theory, informs us that the right optic tract is distributed to, or is in connection with the right half of each eye; *i. e.* the temporal side of the right, and the nasal side of the left eye; and that the left optic tract is in connection with the temporal half of the left and the nasal half of the right retina. Our patient is blind towards his left side, consequently that portion of the retina which should receive the images of external objects from that side is the part affected. The right optic tract is in communication with these portions, therefore this tract is the one involved and incapacitated for transmitting the visual impressions received upon those portions of the retina with which it is in anatomical relation. Pressure is the more probable cause and a pressure that is capable of remission.

Again, our patient has suffered from temporary impairment of sensation and motion in the left side and extremities. Since the decussation of the nerves of sensation and motion takes place in the medulla oblongata, it follows that this paresis is due to some lesion to the right optic thalamus and to the right corpus striatum. This is doubtless in consequence of pres sure in which the right optic tract is involved, as in one common cause; and this pressure may be occasioned by a tumor or by a serous or hemorrhagic effusion. If to a tumor, it may be, and to my mind it is, caused by a gummy tumor, a sequence of the specific virus with which he was affected twenty years before, or possibly by a syphilitic node. The symptoms manifested are consistent with this diagnosis, for it is well known that if a cerebral tumor is very slow of development, the brain substance, and also the nerves may accommodate themselves to its growth, and there may only periodically arise such compression of the vessels at the base of the brain, which, setting up disturbance in the intra-cranial circulation, will give rise to ephemeral hemiplegia, ischæmia and fainting or epileptoid fits. But symptoms of paralysis of the cerebral nerves may supervene, if the tumor pervades, irritates or presses upon the nerve substance, or if the vessels become compressed and the nutrition of the nerves impaired.

Hemorrhagic or serous effusions into the third ventricle, or at the origin of the optic nerve, from the optic thalamus may produce the same effects. One of these effusions *may* have occasioned the phenomena presented by our patient. The cerebral substance adjacent having accommodated itself to the slight pressure produced, or absorption having rapidly taken place, the symptoms presented subsided. Upon these interpretations of the symptoms my treatment was predicated; the removal by absorption of the cause of pressure, whether from a gummy tumor, the consequence of syphilis, from passive effusion, blood or serum from basilar periostitis; from effusion, the product of basilar meningitis or tumor of the pituitary gland.

Hemiopia, as before intimated, *may* also be the result of softening of the brain, occasioned by an atheromatous condition of the basilar arteries. Hemiopia being but a symptom, the treatment must be specially directed to the primary cause, remedial means of a tonic character must be vigorously prosecuted. In this case full doses of pot. iod. were administered alone and in combination with pot. brom., and subsequently hydg. bichl., Turkish baths, and the use of galvanism. Abstinence from the use of alcoholic liquors and of tobacco, also from all sources of excitement incident to the conduct of his business; avoidance of all circumstances calculated to excite passion, anger or resentment; and for the purposes of respite or recreation, recommended a journey to Europe or to California. By these agencies, continued during the period of two months, the area of vision, at *fifteen* inches distant, increased from three and a half inches to sixteen inches, a result highly satisfactory and encouraging.

As regards vision, the prognosis is in all cases extremely unfavorable, for in spite of the best directed efforts, the hemiopic darkening of the field usually progresses in a relatively short time to complete amaurosis. But if the hemiopia on the same side of both eyes is not accompanied by atrophy of the corresponding half of the optic papilla, it is to a certain extent favorable; that is, it is seldom followed by complete blindness, especially when it has existed for some time unchanged. Stellwag states, "It is not uncommon to see a partial or entire clearing up of the darkened portions of the visual field. It is even observed, although rarely, in tumors of the base of the brain and of the cerebral structures. It is quite frequent in cerebral hemorrhages. Such an amaurosis in the beginning, generally extends over the whole tract of one or both of the roots of the optic nerve, with the absorption and shrinking of the clot, it generally diminishes, and is generally limited to the half of one or both retinæ or less, or may even wholly disappear."

No. 620 Locust St.

